# Peer Review of “Converting Organic Municipal Solid Waste Into Volatile Fatty Acids and Biogas: Experimental Pilot and Batch Studies With Statistical Analysis”

**DOI:** 10.2196/69896

**Published:** 2025-02-04

**Authors:** Dina Elsalamony

**Affiliations:** 1 Department of Biotechnology, Institute of Graduate Studies & Research, University of Alexandria, Alexandria, Egypt

**Keywords:** multistep fermentation, specific methane production, anaerobic digestion, kinetics study, biochar, first-order, modified Gompertz, mass balance, waste management, environment sustainability

*This is a peer-review report for “Converting Organic Municipal Solid Waste Into Volatile Fatty Acids and Biogas: Experimental Pilot and Batch Studies With Statistical Analysis.”*

## Round 1 Review

### General Comments

Generally, the manuscript [1] should be strictly improved in English language writing and corrected for all grammatical errors throughout the whole manuscript. The author has to use a uniform style of the English language, either American or British English. Further English assistance is particularly required. Many missing articles and a lot of grammatical and punctuation errors must be corrected in the manuscript as in the corrected abstract.

### Specific Comments

This paper shows an important aspect of multiple fermentation steps for the complete utilization of municipal solid waste and conversion to useful products, which is highly recommended for circular economic sustainability worldwide. However, it needs some major revision and arrangement to allow for a better presentation of this valuable work.

#### Major Comments

##### Title

1. “Valorization of Organic Fraction of Municipal Solid Waste Through Production of Volatile Fatty Acids (VFAs) and Biogas” is a long title that should be shortened to be more concise with no abbreviations—more indicative. Suggested title: “Valorization of Organic Municipal Solid Waste for Volatile Fatty Acids and Biogas Production.”

##### Abstract Section

2. Generally speaking, it must be more concise and specific.

3. Please clearly mention the take-home message and the main findings of the research.

4. The abstract is too long and lacks the main methodology and main experimental techniques that were carried out in this work. The author may add some hints about the main methods used before mentioning the main results.

##### Manuscript

5. Keywords: Words must be modified to be more informative and representative of the research interest and differ from the word in the manuscript title. Maybe add “Multi Step of Fermentation Process” or “Waste Management and Environment Sustainability.”

6. Arrangement of the experimental work in the manuscript may be needed in the Results and Discussion accordingly.

7. There is a lack of figures to describe the main parameter optimization steps well. Please reformulate to describe some data using figures with error bars.

8. The SD and table footnotes with the number of replicates should be noted underneath all of the given tables.

9. A mechanistic in-detail discussion is required, not just comparing your results with the previous work; justify better.

10. In research articles, do not include any table comparing literature results; the author can discuss the main findings in the text itself, as in Table 5.

11. The Conclusions section is missing in the manuscript to summarize and point out the novelty and the main findings from the research.

12. Generally speaking, in academic writing, (1) abstracts do not include abbreviations, (2) avoid articles in the title (the, a, an), and (3) avoid keywords that exist in the title.

13. As a rule of thumb, no dots in titles or subtitles as in the Experimental section, Anerobic Pilot Unities, etc.

14. Multiple references should be merged, not written separately, as in “29, 30” and “23, 27”; the author may use the merge reference option in reference software.

15. The author may add numbers for all titles and subtitles accordingly all over the manuscript.

### Minor Comments

16. The author should avoid general and well-known information, and be selective in the recent references used. May add one small paragraph to the Biological Waste Management and Environment Sustainability section.

17. The author should clarify the main aim of the work clearly in the last paragraph of the Introduction.

18. Do not use our, we, or us in academic writing.

19. The author may mention novel applications of VFA and biogas. Mention different novel sources of biogas production.

20. The author should mention the gas chromatography type, gas injection rate, column dimensions, and the used carrier gas in the main document.

21. The author did not mention that flushing with nitrogen or carbon dioxide took place in anaerobic digestion while feeding reactors and how the anaerobic conditions were maintained; please mention it clearly or add the references used for the methodology.

22. Organize titles all over the manuscript.

23. Generally, the subtitles are too generic; modify them to be more indicative and precise.

24. “unless Saturday and Sunday” in line 208 is not important information; the suggested word “daily” is enough.

25. “Unite”: Please correct.

26. Remove the grid lines in the figures.

27. The author has to mention the range used for the chemical oxygen demand method, and the original reference should be cited appropriately.

28. “As can be seen”: This statement is repetitive more than once in the Discussion, in lines 301, 315, and 423.

29. Figure 3 caption: mesophilic fermentation: Please specify which stage because both of the sequential steps were called mesophilic fermentation in Figure 1.

30. What is the rationale for comparing 3 days to 4.5 days for all the used systems; the author may justify why 4.5 days is better to complete with this hydraulic retention time in the rest of the experiments or describe the one variable at a time optimization method that is used to determine the significant factors and the insignificant one; mention them clearly. Also, use in the Discussion the terms “significant” and “insignificant” according to the obtained *P* value.

31. The author has to mention tables and figures in the text in their appropriate place.

## Round 2 Review

This paper is greatly enhanced compared to the previous copy, and the author followed the previous comments precisely.

I recommend its publication. Thanks for allowing me to review this interesting work.

### General Note

The Word file is the correct revised one, but the PDF seems to be the old version.

## Abbreviations

VFA: volatile fatty acid
